# Changes in frailty status and discharge destination post emergency laparotomy

**DOI:** 10.1186/s13017-025-00612-8

**Published:** 2025-04-25

**Authors:** Hwei Jene Ng, Nicholas J. W. Rattray, Tara Quasim, Susan J. Moug

**Affiliations:** 1 Department of General Surgery, Royal Alexandra Hospital, NHS Greater Glasgow and Clyde, Corsebar Road, Paisley, PA2 9PN UK; 2 College of Medical, Veterinary and Life Sciences, University of Glasgow, University Avenue, Glasgow, G12 8QQ UK; 3 Strathclyde Institute of Pharmacy and Biomedical Sciences, University of Strathclyde, 161 Cathedral Street, Glasgow, G4 0RE UK; 4 Department of Critical Care, Glasgow Royal Infirmary, NHS Greater Glasgow and Clyde, 84 Castle Street, Glasgow, G4 0SF UK; 5 Department of Colorectal Surgery, Golden Jubilee Hospital, NHS Waiting Times, Agamemnon Street, Clydebank, G81 4DY UK

**Keywords:** Emergency laparotomy, Frailty, Discharge destination

## Abstract

**Background:**

Pre-operative frailty adversely affects morbidity and mortality after emergency laparotomy (EmLap), especially in older adults (65 years and above). Little is known about frailty after EmLap. We explored the change in frailty status from pre- to post-EmLap and any influence on discharge destination.

**Methods:**

EmLap patients aged ≥ 65years from an acute surgical site were recruited from May 2022 to April 2023. Prospective data collection included demographics, frailty, mortality and discharge destination. Frailty was assessed using the Rockwood Clinical Frailty Scale at pre-EmLap and day-90 post-EmLap (< 4 as non-frail, 4 as pre-frail and > 4 as frail). EmLap patients with no 90-day follow-up were excluded. A p-value of < 0.05 was considered significant.

**Results:**

63 EmLap patients were included in the study. The median age was 75 years (range 65–91 years) with 36 (57.1%) females. Eleven (17.5%) were living with frailty pre-EmLap, and 10 (15.9%) developed new frailty by day-90 post-EmLap. Pre-EmLap, all patients came from home with 20.6% of the frail and pre-frail group having a package of care service (POC) in place. On 90-day post-EmLap, 1 was still an inpatient but 25.8% had a change in discharge destination: care home (*n* = 1), home with new POC (*n* = 2) and home with increased POC (*n* = 13). Of the 16 patients with change of discharge destination, 9 (56.3%) were frail pre-EmLap. There was a significant association between pre-EmLap frailty and change in home circumstances on discharge (*p* < 0.00001).

**Conclusions:**

Emergency surgery can increase a patient’s frailty status and significantly increases care requirements and social support after hospital discharge. Frailty assessment needs to be performed before and after admission in all EmLap patients to improve post-EmLap care planning and patient expectations.

## Introduction

The population structure of the United Kingdom (UK) is currently transitioning towards a higher proportion of older adults [1]. According to the Office of National Statistics, the projected number of people aged 65 and above will be over 19 million by 2050, representing almost 24% of the total population. Currently, in Wales and England, 30.1% of those aged 65 years and above (3.3 million) are living alone [1]. From a surgical perspective, the implication of this growing number of older adults is reflected in the National Emergency Laparotomy Audit (NELA) and the Emergency Laparoscopic and Laparotomy Scottish Audit (ELLSA) [2, 3]. Both these audits collect emergency laparotomy and laparoscopic (EmLap) data in the UK and the most recent reports published from both audits stated that more than half of the EmLap patients were 65 years and above.

There is no standardised definition of frailty but it can be described as ‘a state of increased vulnerability to illnesses or acute stressors due to multisystem aging related physiological decline’ [4]. Frailty is strongly correlated to age but not directly caused by it [5]– indicating a range of underlying factors that have the potential to be assessed and interacted with, to enable more precision based clinical treatment. In older adults, rates of morbidity and mortality after emergency surgery are found to be higher than younger cohorts, and the risk of mortality was also found to double when frailty is present [2]. In NELA, the mortality risk is considered high when the NELA mortality risk is 5% and above. However, due to the significant impact of frailty on the EmLap outcomes, frail patients are now considered ‘high risk’ despite having a low calculated NELA mortality risk [2].

Out with large national databases, recent studies have shown that EmLap can induce or increase frailty [6, 7] and as a consequence, significantly impact a patient’s quality of life [8]. For surviving post-EmLap patients living with frailty, many experience a loss of independence or baseline function, often requiring long term residential care [6]. However, the literature on post-EmLap outcomes, especially regarding discharge destination from hospital remains scarce [9]. Most of the consenting process for EmLap focuses primarily on post-operative survival and complications, with limited emphasis on the effects on independence or function [10]. As a result, shared decision making is often lacking, and this oversight can lead to decisional regret, in particular among frail post-EmLap patients [11].

The aim of this study was to explore the changes of frailty status at 90-day post-EmLap and to investigate the association between pre-EmLap frailty status and changes in discharge destination.

## Methods

This study was a prospective, single-centred, observational study on consecutive patients who underwent emergency laparotomy (EmLap) from May 2022. It was part of a larger trial, approved by the local and national ethics committee (Scotland A Research Ethics Committee, IRAS 293392) and was registered at http://www.clinicaltrials.gov (NCT05416047). The study protocol of the trial has been published [12] and the recruitment completed in April 2023.

In the study protocol [12], all consecutive EmLap patients were evaluated for inclusion and exclusion criteria as per NELA criteria. Patients who lacked capacity to consent but were eligible for the study were included with consent obtained from their next of kin or welfare attorney. For this study, only patients aged ≥ 65 and above with full 90 days follow up were included. EmLap patients ≤ 65 years old and those with incomplete 90-day follow-up were excluded.

Baseline demographic data included: co-morbidity that was assessed using Charlson Co-morbidity Index [13] (CCI) with score ≤ 2 as mild, score 3–4 as moderate and score ≥ 5 as severe; admission source (home independently or home with package of care or care home); American Association of Anaesthesiologists physical status classification (ASA) score; frailty status (The Rockwood Clinical Frailty Scale (CFS) [14] was used to assess frailty, with score < 4 as non-frail, 4 as pre-frail and > 4 as frail); complications (evaluated using the Clavien-Dindo Classification [15] up to day 30); and discharge data (discharge destination included discharge home independent, discharge home with new or increase package of care or care home). At 90-days post-EmLap, frailty status (assessed by research team as part of the larger trial) and discharge destination were collected.

Categorical data such as demographic, peri-operative and post-operative measures were expressed as numerical numbers and percentages while continuous data was expressed as median values. Fisher’s Exact test was used to correlate frailty status and discharge destination, and logistic regression was performed for variables in predicting change of home environment. Receiver Operating Characteristic (ROC) curve analysis was performed to assess the sensitivity and specificity of the frailty status in predicting discharge destination. A p-value of < 0.05 was considered significant. The statistical analysis was performed on IBM SPSS Statistics for Windows Version 29.0.1, released 2023.

## Results

### Patient characteristics

A total of 87 patients aged ≥ 65 underwent emergency laparotomy during the 12-month recruitment period. The analysis for this study was based on 63 patients after excluding 24 patients with incomplete follow- up (8 lost to follow- up and 16 from mortality). Among these 16 mortalities, 11 occurred within 30 days (10 as inpatients) while 5 occurred between 30 and 90 days with only 1 of the 16 mortalities (6.3%) classified as frail.

Of the 63 analysed patients, 57.1% were female and the median age was 75 years (range: 65–91 years). The majority of patients had a CCI score of 3 (34.9%) and ASA score of 3 (43%). Pre- EmLap, 17.5% were classified as frail, 57.1% pre-frail and 25.4% non-frail. 20.6% were admitted from home with package of care (POC) already in place (6 of the frail and 7 of the pre-frail group). The main indications for EmLap were small bowel obstruction (47.6%) and lower gastrointestinal perforation (15.9%) with 25.4% underwent adhesiolysis. There were 16 (25.4%) who had EmLap due to colorectal cancer. The demographic data and peri-operative data are shown in Table 1.

**Table 1:** The baseline demographic and peri-operative data in this cohort of emergency laparotomy patients aged 65 years and above with complete 90-day follow up. ASA; American association of anaesthesiologists physical status classification. CFS; Rockwood clinical frailty scale. EmLap; emergency laparotomy

Characteristics	Number (%)
**Sex**	
Male Female	27 (42.9%) 36 (57.1%)
**Charlson Co-Morbidity Index Score**	
Mild (≤ 2) Moderate (3–4) Severe (≥ 5)	5 (8.0%) 36 (57.1%) 22 (34.9%)
**ASA**	
1 2 3 4 5	0 20 (31.7%) 27 (42.9%) 15 (23.8%) 1 (1.6%)
**CFS pre-EmLap**	
< 4 (non-frail) 4 (pre-frail) > 4 (frail)	16 (25.4%) 36 (57.1%) 11 (17.5%)
**Admission source pre-EmLap**	
Home independent Home with package of care Care home	50 (79.4%) 13 (20.6%) 0
**Indication for EmLap**	
Small bowel obstruction Lower gastrointestinal perforation Large bowel obstruction Anastomotic leak Incarcerated hernia containing bowel Acute mesenteric ischemia Upper gastrointestinal perforation Fistula Colitis	31 (47.6) 10 (15.9%) 9 (14.3%) 4 (6.3) 3 (4.8%) 3 (4.8%) 2 (3.2%) 1 (1.6%) 1 (1.6%)
**Type of EmLap procedure**	
Adhesiolysis Right colectomy Small bowel resection Hartmann’s procedure Subtotal colectomy Resection of anastomosis Omental patch repair Enterolithotomy Defunctioning colostomy Thrombectomy of superior mesenteric artery	16 (25.4%) 13 (20.6%) 10 (15.9%) 10 (15.9%) 4 (6.3%) 4 (6.3%) 2 (3.2%) 2 (3.2%) 1 (1.6%) 1 (1.6%)

### Post-EmLap complications and readmission within 30 days

The median length of hospital stay post-EmLap was 12 days (ranged 2 to 106 days). 41.3% experienced complications within 30 days post-EmLap. There were 2 patients that required a further operative intervention (1 for washout and drain insertion post resection of anastomosis and 1 for refashioning of stoma post Hartmann’s procedure).

7.9% had readmission to hospital within 30 days post-EmLap. The indications were acute kidney injury from high stoma output (2 patients), post-operative collections needing intravenous antibiotics (2 patients) and melaena which resolved with conservative management (1 patient).

### 90-day follow-up

**Fig. 1:**
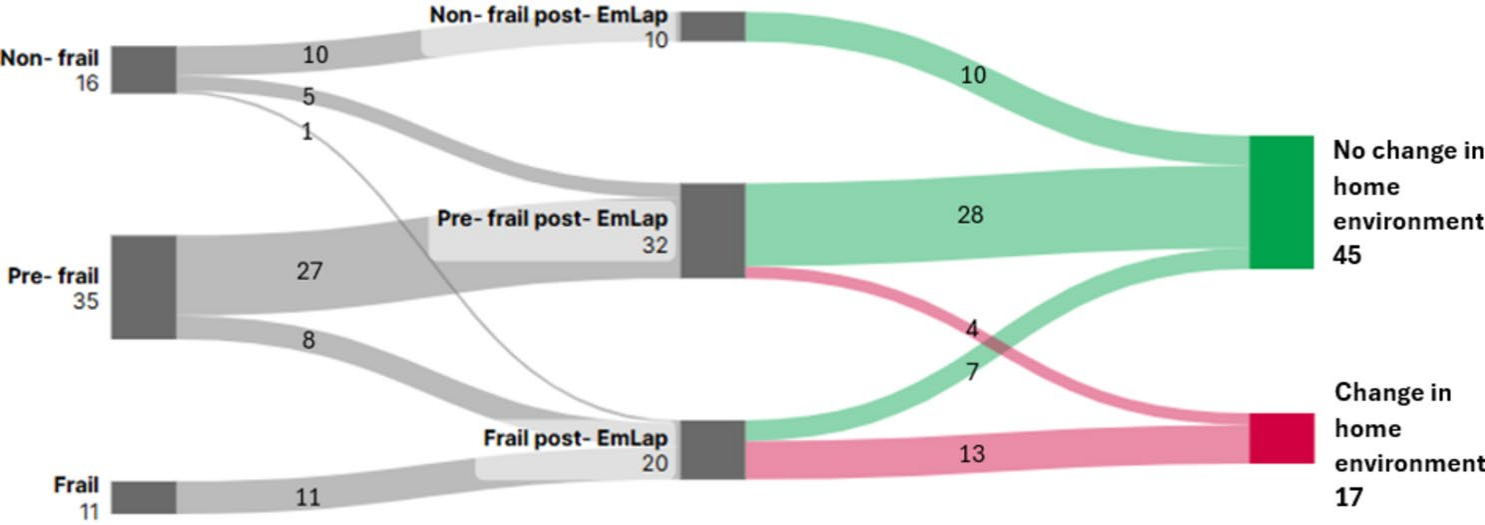
The Sankey chart showing changes in patients’ (*n* = 62) frailty status at pre- and post-EmLap and home environment after discharge

On 90-day post-EmLap, frailty scores increased with 33.3% of patients now classified as frail (versus 17.5% pre-EmLap). Of these, 10 (15.9%) had developed new frailty (1 from non-frail and 9 from pre-frail). One patient was still an inpatient at 90-day post-EmLap, therefore 62 patients discharge destination were explored. It showed 17 (27.4%) had a change of home environment post discharge. The change in home environment and frailty status is illustrated in Fig. 1. The follow-up data up to 90-day is detailed in Table 2.

**Table 2:** The post emergency laparotomy (EmLap) data including follow- up data up to day 90. CFS; Rockwood clinical frailty scale. CD; Clavien Dindo classification

Characteristics post-EmLap (*n* = 63)	Number (%)
**Post-operative hospital stay (day)**	
≤ 10 11–30 3–60 ≥ 61	27 (42.9%) 26 (41.2%) 7 (11.2%) 3 (4.8%)
**30-day morbidity**	
None CD1 CD2 CD3 CD4a	37 (58.7%) 7 (11.1%) 16 (25.4%) 2 (3.2%) 1 (1.6%)
**30-day readmission rate**	
Yes No Still inpatient	5 (7.9%) 48 (76.2%) 10 (15.9%)
**CFS post-EmLap day-90**	
< 4 (non-frail) 4 (pre-frail) > 4 (frail)	10 (15.9%) 32 (50.8%) 21 (33.3%)
**Discharge destination post-EmLap (** ***n*** ** = 62)**	
Home independent Home with increased package of care Home with new package of care Care home	46 (74.2%) 13 (21.0%) 2 (3.2%) 1 (1.6%)

On day-90 post-EmLap, all non-frail patients (CFS score ≤ 3) at pre-EmLap (16 patients, 25.4%) and post-EmLap (10 patients, 15.9%) had no change to home environment or discharge destination 90-day post-EmLap. Of the 16 patients with change of discharge destination, 9 (56.3%) were frail at pre-EmLap. There were significant associations between pre- and post-EmLap frailty and change in home circumstances post discharge (*p* < 0.001). Other variables including age, sex, ASA score, comorbidities and 30-day morbidity were not significantly associated to change in home environment (*p* > 0.05). When compared to frail patients, the likelihood of pre-frail patients having a change in discharge destination is lower with odds ratio of 0.056 at pre-EmLap and odds ratio of 0.095 at post-EmLap. Comparison of home environment pre- and post-EmLap with frailty status pre-EmLap are shown in Table 3.

**Table 3:** Analysis between change in home environment post discharge after emergency laparotomy (EmLap) with pre- frail and frail patients at pre- and post-EmLap. CI; confidence interval

Frailty status	No change to home environment post discharge	Change of home environment post discharge	Odds ratio (CI)	*p*-value
**Pre-EmLap**				*P* = 0.001
Pre-frail Frail	28 (80.0%) 2 (18.2%)	7 (20.0%) 9 (81.8%)	0.056 (0.010–0.317)	
**Post-EmLap**				*P* < 0.001
Pre-frail Frail	28 (87.5%) 8 (40.0%)	4 (12.5%) 12 (60.0%)	0.095 (0.024–0.378)	

**Fig. 2:**
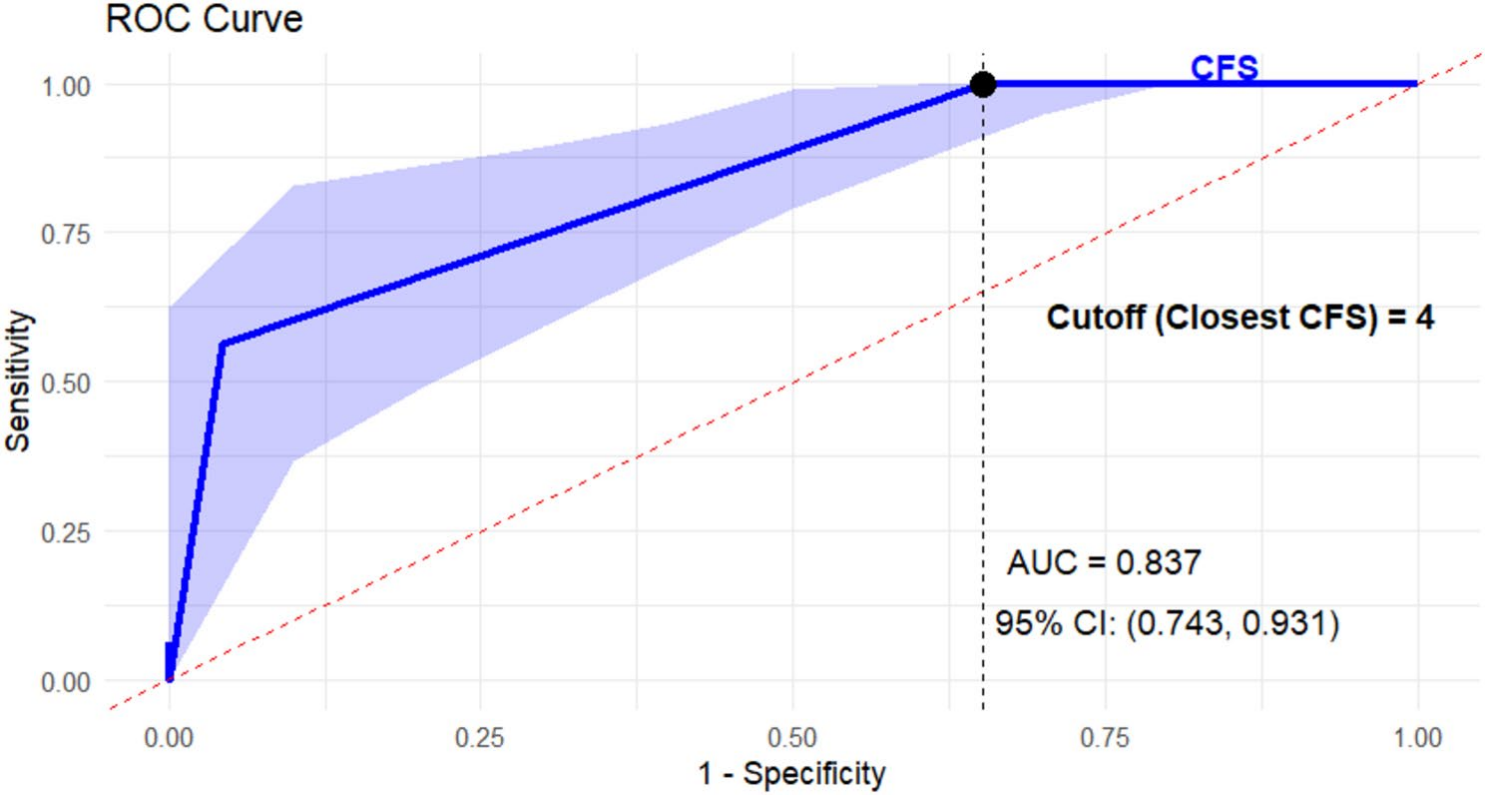
The receiver operating characteristic (ROC) curve analysis demonstrated that the Clinical Frailty Scale had good discriminatory ability in predicting post-operative discharge destination changes

The receiver operating characteristic (ROC) curve analysis demonstrated that frailty had good discriminatory ability in predicting post-operative changes in discharge destination, with the area under the curve (AUC) of 0.837 (95% CI: 0.743–0.931, *p* < 0.001). The cutoff CSF score of 4 pre-EmLap was the best overall threshold for predicting post-operative change in discharge destination based on frailty status (Youden index was 0.519). This indicated that frailty score of 4 and above is a significant predictor of discharge destination changes (Fig. 2).

## Discussion

This study is the first to assess how frailty status changes up to 90 days post emergency laparotomy (EmLap). We report on surgical induced frailty, where 15.8% of the study participants aged 65 years and above developed new frailty 90-day post-EmLap (increasing from 17.5% pre-EmLap to 33.3% post-EmLap). The explicit pathophysiology to cause this remains unknown. However, it is well established that frailty increases the risks of poor outcomes post- EmLap, including mortality, length of hospital stay post- EmLap and 30-day readmissions [16]. Therefore, frailty should be identified early at all stages of surgical assessment, as well as pre-EmLap. However, recognising that frailty scoring pre-EmLap may be missed in emergency settings, frailty assessment should still be performed post-operatively. In fact, the Emergency Laparotomy Enhanced Recovery After Surgery Society [17] published their guideline (part one) in 2021, with their 4th recommendation revolved around the importance of risk assessment at pre- and post-operative periods. They found missed scoring of frailty reduced the protective peri-operative care, such as multidisciplinary communication on patient care and planned critical care admissions, leading to worse outcomes compared to patients with early frailty assessment [17]. These findings underscore that frailty assessment at peri-EmLap period is crucial, as it ensures early, comprehensive peri-operative care (e.g. timely geriatric team involvement) for EmLap patients which ultimately would enhance patient’s overall outcomes.

Few studies have explored discharge destinations post-EmLap in frail patients. Kennedy et al. [18] conducted a systematic review and meta-analysis on the impact of frailty on post emergency general surgery outcomes from 2009 to 2019, and only found six studies, of which only three included the discharge destination. Their studies showed that frail patients were significantly more likely to be discharged to rehabilitation centres or nursing homes compared to the non-frail patients. Similarly, Carter et al. [9] found 37.4% of patients required an increased level of care post-EmLap, with pre-operative frailty being a greater predictive power than age. Interestingly, similar results were found in elective surgical settings, as shown by Robinson et al. [19] where 30% of patients aged ≥ 65 years who underwent major elective surgery and required post-operative admission to intensive care were discharged to an institute besides home. In this study, frail patients (CFS score > 4) were observed to have a higher incidence of changes in home environment or discharge destination, with 26% of the participants requiring a transition in living arrangements. Given these findings, patient centred discharge planning should be prioritised for frail older EmLap patients, alongside efforts to improve the standard post-operative outcomes. Discussions surrounding post-EmLap changes in home environment should be incorporated into the pre-EmLap consenting process to ensure informed consent and shared decision making. In addition, patient’s wishes should be considered, and post discharge support should be arranged with the goal of gaining back independence. These factors may be more valuable for patients, as quality of life post-EmLap have been shown to be significantly affected, especially in frail individuals [8, 20].

In this study, 25% of the pre- frail patients developed frailty post-EmLap. While there is limited high level evidence demonstrating the benefits of early mobilisation and nutrition in post-EmLap patients, these interventions are strongly recommended in the perioperative care for EmLap patients guidelines, proposed by the Enhanced Recovery After Surgery (ERAS^®^) Society [21]. However, these recommendations are primarily based on level 1 evidence or evidence from elective surgeries. Gené Huguet et al. [22] performed a randomised clinical trial comparing standard primary healthcare management with an intervention group (physical intervention, dietary and polypharmacy review) in pre-frail participants aged ≥ 80 years living in the community. They found that the intervention group had a lower frailty status 1 year later. Similarly, Lawrence et al. [23] found that functional decline was highest during the first week post major elective surgery. These findings provide valuable insights into the best timing for post-EmLap interventions and care. A multidisciplinary approach, especially with input from the geriatric team during the post-EmLap care, is crucial in optimising post-EmLap outcomes [24]. Correcting nutritional deficits and promoting early mobilisation as part of patient-oriented rehabilitation post-EmLap can aid in reducing muscle wasting and functional decline, hence improving or postponing progression of frailty. These interventions are key to reduce loss of functional independence post-EmLap [21] and should be integrated into standard post-EmLap care.

The primary limitations of this study include a small sample size and a single centre design, which may limit the generalisability of the findings to the broader population. Another limitation is the assessment of frailty status at 90-day post-EmLap, which may not capture the full extent of longer-term changes to frailty after emergency laparotomy. Additionally, the study did not explore the financial burden on relatives providing unpaid care, especially when patients were discharged home without a formal care package and required assistance from relatives. Nonetheless, conducting well- designed research in the emergency laparotomy setting is challenging. Despite these constraints, this study contributes to the limited body of studies looking into the impact of frailty in EmLap patients and post discharge outcomes. Further research involving multi-centre studies with larger sample sizes and longer follow-up periods are required to validate the findings in this study.

## Conclusion

Emergency surgery increases post-operative frailty in the older adult population (17.5% versus 33.3% post-EmLap). This surgical-induced frailty is associated with over a quarter of patients requiring more social support and care after hospital discharge. All older adults undergoing emergency surgery should have frailty scoring before and after their admission to optimise care planning and patient expectations.

## Data Availability

No datasets were generated or analysed during the current study.

## Declarations

### Further information

The abstract was submitted and presented as oral presentation in the Association of Surgeons of Great Britain and Ireland Conference 2024. DOI: 10.1093/bjs/znae197.100.
